# Proposal of minimum elements for screening and diagnosis of gastric cancer by an international Delphi consensus

**DOI:** 10.1002/deo2.97

**Published:** 2022-02-24

**Authors:** Naomi Kakushima, Mitsuhiro Fujishiro, Shannon Melissa Chan, George Adel Cortas, Mario Dinis‐Ribeiro, Robinson Gonzalez, Shinya Kodashima, Sun‐Young Lee, Enqiang Linghu, Katsuhiro Mabe, Wensheng Pan, Adolfo Parra‐Blanco, Mathieu Pioche, Antonio Rollan, Kazuki Sumiyama, Miguel Tanimoto, World Endoscopy Organization Stomach and Duodenal Diseases Committee

**Affiliations:** ^1^ Department of Gastroenterology and Hepatology Nagoya University Graduate School of Medicine Aichi Japan; ^2^ Department of Surgery Prince of Wales Hospital The Chinese University of Hong Kong Shatin Hong Kong; ^3^ Saint George Hospital University Medical Center Faculty of Medicine University of Balamand Beirut Lebanon; ^4^ Department of Gastroenterology Portuguese Oncology Institute of Porto Porto Portugal; ^5^ Medical School Pontificia Catholic University Santiago Chile; ^6^ Department of Medicine Division of Gastroenterology School of Medicine, Teikyo University Tokyo Japan; ^7^ Department of Internal Medicine School of Medicine Konkuk University Seoul Korea; ^8^ Department of Gastroenterology and Hepatology The First Medical Center of Chinese PLA General Hospital Beijing China; ^9^ Junpukai Health Maintenance Center Kurashiki Okayama Japan; ^10^ Department of Gastroenterology Zhejiang Provincial People's Hospital People's Hospital of Hangzhou Medical College Hangzhou China; ^11^ NIHR Nottingham Biomedical Research Centre Nottingham University Hospitals NHS Trust and the University of Nottingham Nottingham UK; ^12^ Department of Gastroenterology and Endoscopy Edouard Herriot Hospital Lyon France; ^13^ Unidad de Gastroenterología Facultad de Medicina Clinica Alemana Universidad del Desarrollo Santiago Chile; ^14^ Department of Endoscopy School of Medicine, The Jikei University Tokyo Japan; ^15^ Ancillary and Diagnosis Services National Institute of Medical Sciences and Nutrition Salvador Zubiran Mexico City Mexico

**Keywords:** diagnosis, gastric cancer, guideline, screening, World Endoscopy Organization

## Abstract

The World Endoscopy Organization Stomach and Duodenal Diseases Committee extracted minimum elements for screening and diagnosis of gastric cancer (GC) in aim to support countries that do not have national guidelines on screening and diagnosis of GC. Current national or international guidelines were collected worldwide and recommendations were classified according to the quality of evidence and were finalized through a modified Delphi method. The minimum elements consist of seven categories: [1] Extraction of high‐risk patients of GC before esophagogastroduodenoscopy (EGD), [2] Patients who need surveillance of GC, [3] Method to ensure quality of EGD for detection of GC, [4] Individual GC risk assessment by EGD, [5] Extraction of high‐risk patients of GC after EGD [6] Qualitative or differential diagnosis of GC by EGD, and [7] Endoscopic assessment to choose the therapeutic strategy for GC. These minimum elements will be a guide to promote the elimination of GC among countries with a high incidence of GC who lack national guidelines or screening programs.

## INTRODUCTION

Gastric cancer (GC) is the sixth most common type of cancer and the fifth leading cause of cancer death worldwide.^1^ In countries with a high incidence of GC, national or community‐based screening programs of esophagogastroduodenoscopy (EGD) have been introduced.^2^, ^3^ Screening EGD has been reported to be an effective method to decrease GC mortality.^2^, ^3^

The role of EGD is essential for the screening, diagnosis, and treatment of GC. A well‐known cause of GC is *Helicobacter pylori* (*H. pylori*) infection.^4^, ^5^ Patients with extensive atrophic gastritis or gastric intestinal metaplasia (GIM) due to *H. pylori* have the highest risk of developing GC.^6^ Risk stratification of candidates is necessary for efficient screening and to decrease the cost and human resources for EGD. Several national or international guidelines for GC diagnosis and treatments have been published in countries with different GC incidences, socioeconomic backgrounds, and accessibility to EGD.

The aim of this study was to extract minimum elements for screening and diagnosis of GC and support countries that do not have national guidelines on this topic.

## METHODS

National and international guidelines of GC diagnosis and treatments were searched by 16 members of the World Endoscopy Organization (WEO) Stomach and Duodenal Diseases Committee in September 2020 and were collected from nine countries or regions. The research was performed on articles published until September 2020 using PubMed and by manual search using the following keywords: “gastric/stomach cancer/carcinoma”, “premalignant or malignant conditions, “prevention”, “screening”, “detection”, “diagnosis”, “management”, and “guideline”. A narrative review of different guidelines was performed by two members, and all statements regarding screening and diagnosis for GC were extracted including countries with multiple guidelines. Statements were re‐classified according to the topic, quality of evidence, and the levels of recommendation, and summarized to provide a minimum requirement. The GRADE system for rating the quality of evidence; high quality, moderate quality, low quality, very low quality, was used.^7^ The summarized statements were circulated to 16 WEO committee members for the level of agreement. The members voted anonymously using a Delphi method and a statement with disagreement was revised until there was no disagreement. The original 5‐point scale developed by Mancuso et al. was modified to a 4‐point scale (Agree, mostly agree, partially agree, disagree) by omitting “this does not apply to me”.^8^ Consensus was obtained using two rounds. After achieving a consensus with no disagreement, final minimum statements were created. The last version was finally agreed upon by the steering committee of WEO.

## RESULTS

### Global collection of guidelines for GC management

Collected guidelines from different countries,^9^, ^10^, ^11^, ^12^, ^13^, ^14^, ^15^, ^16^, ^17^, ^18^, ^19^ as well as the incidence and mortality of GC are shown in Table 1 (available on the website of GLOBOCAN, http://globocan.iarc.fr/). According to the incidence of GC (age‐standardized ratio per 100,000), countries were classified into: very high: 11.0<, high: 7.4–11.0, moderate: 5.2–7.4, low: 3.5–5.2, and very low: <3.5. In countries with a very high incidence of GC, guidelines have been established in only a few countries with updated versions. In Japan and South Korea, where a national mass screening program for GC is available, the mortality compared to the incidence was lower than countries with a high incidence with no GC guideline or screening program.

**TABLE 1:** Presence of guidelines, incidence, and mortality of gastric cancer from different countries in the world (collected by September 2020)

**Country**	**Incidence (ASR)**	**Mortality (ASR)**	**Presence of GL**	**committee**	**First published year**	**Latest version, publication year**	**Language**
Mongolia	32.5	24.6	No	–	–	–	–
Japan	31.6	8.2	Yes	Japanese Gastric Cancer Association, Japanese Society of Gastroenterological Endoscopy	1962	Classification: 15th ed., 2017 Guideline: 5th ed., 2018^11^ Diagnosis: Dig Endosc, 2020^18^	Japanese/English
Korea	27.9	11.9	Yes	Korean Gastric Cancer Association and others	2004	J Gastric Cancer, 2019^13^ Endoscopic treatment: Clin Endosc, 2020	Korean/English
Tajikistan	23.4	19.7	No	–	–	–	–
China	20.6	15.9	Yes	Chinese Society of Clinical Oncology	2005	Cancer Commun, 2019^14^ Endoscopic treatment: Chi J GI Endosc, 2019	Chinese/English
Chile	13.1	10.0	Yes	Chilean Association for Digestive Endoscopy	2014	Rev Med Chile, 2014^9^	Spanish
Portugal	11.0	7.9	Yes	European Society of Gastrointestinal Endoscopy, Sociedade Portuguesa de Endoscopia Digestiva and others	2012	Endoscopy, 2019^16^	English
World	11.1	7.7	No	–	–	–	–
Mexico	6.2	4.7	Yes	Asociacion Mexicana de Gastroenterologia	2020	Revista de Gastroenterologia de Mexico, 2020^19^	Spanish/English
France	4.7	2.9	Yes	French Society of Gastroenterology, Societe Francaise d'Endoscopie and others	2016	Dig Liver Dis, 2018^12^	French/English
The USA	4.2	1.7	Not specifically	American Gastroenterological Association American Society for Gastrointestinal Endoscopy		Gastroenterology, 2020^17^ Gastrointest Endosc, 2015^10^	English
The UK	4.0	2.4	Yes	British Society of Gastroenterology and others	2002	Gut, 2019^15^	English

ASR: Age‐standardized ratio per 100,000. Very High: 11.0<, High: 7.4–11.0, Moderate: 5.2–7.4, Low: 3.5–5.2, Very low: < 3.5, based on GLOBOCAN 2020 (Cancer Today, International Agency for Research on Cancer, data accessed on Feb 27, 2021, http://globocan.iarc.fr/). GL: guideline.

**TABLE 2:** Summary of recommendations

	**Level of evidence**
Patients with *H. pylori* infection and chronic atrophic gastritis or GIM are at risk for gastric adenocarcinoma.	High quality
A combination of serum *H. pylori* antibody and pepsinogen level may be useful to stratify patients in need of endoscopy.	Low quality
Surveillance endoscopic examination is recommended for patients with risk factors for gastric cancer.	Moderate quality
The stomach should be systematically observed with sufficient time to detect gastric cancer.	Moderate quality
In combination with white light endoscopy, IEE and biopsies should be considered for the assessment of chronic atrophic gastritis and GIM.	Moderate quality
Extensive atrophy or presence of GIM should be identified by endoscopic findings or histology.	Moderate quality
High definition endoscopy with chromoendoscopy, IEE, and where available, magnification endoscopy is recommended for the diagnosis of neoplastic lesions.	High quality
Patients with an endoscopically visible lesion harboring dysplasia or carcinoma should undergo endoscopic staging and treatment.	Moderate quality

Abbreviations: GIM, gastric intestinal metaplasia; IEE, image enhanced endoscopy.

### Extraction of statements regarding screening and diagnosis for GC

Based on the collected guidelines, two members performed an initial review to extract statements regarding screening and diagnosis for GC. In total, 79 statements were extracted and classified into seven categories: extraction of high‐risk patients of GC before EGD (10 statements), patients who need surveillance of GC (16 statements), a method to ensure the quality of EGD for detection of GC (12 statements), individual GC risk assessment by EGD (23 statements), extraction of high‐risk patients of GC after EGD (four statements), qualitative or differential diagnosis of GC by EGD (five statements), and endoscopic assessment to choose the therapeutic strategy for GC (nine statements). (Table S1)

### Summary of statements and consensus among WEO Stomach and Duodenal Diseases Committee (Table 2)

1. Extraction of high‐risk patients of GC before EGD

Most guidelines commented on several risk factors such as *H. pylori*, gastric atrophy, GIM, hereditary disease, smoking, and other possible factors including diet, lifestyle preferences, and Epstein‐Barr Virus infection. In moderate to high incidence countries, identification or stratification of patients at high risk is an efficient way to perform screening. However, issues about the optimal method for risk stratification still remain. The age to start screening differed among countries; 40 years (South Korea^13^), symptomatic patients over 40 years (Chile^9^), individuals aged ≥ 50 years with multiple risk factors (UK^15^), individuals aged ≥ 50 years (Japan), and in France^12^ depending on the age of asymptomatic individuals with 1st‐degree relative history of cancer; by a urea breath test or serology for *H. pylori* before 45 years old or by gastroscopy with biopsies over 45 years old.

The integrated statements in this category were;

- **Patients with *H. pylori* infection and chronic atrophic gastritis or GIM are at risk for gastric adenocarcinoma**.

(High quality evidence; Agree: 77%, Mostly agree: 23%, Partially agree: 0%, Disagree: 0%)

- **A combination of serum *H. pylori* antibody and pepsinogen (PG) level may be useful to stratify patients in need of endoscopy**.

(Low quality evidence; Agree: 50%, Mostly agree: 33%, Partially agree: 17%, Disagree: 0%)

2. Patients who need surveillance of GC

Surveillance gastroscopy is recommended for patients with atrophic gastritis or GIM, and the surveillance interval should be adjusted to the estimated risk of GC. Patients who have had a curative endoscopic resection for early GC are recommended for regular surveillance (Japan,^18^ Europe,^16^ UK,^15^ China,^14^ and South Korea^13^).

The integrated statement in this category was:

- **Surveillance endoscopic examination is recommended for patients with risk factors for GC**.

(Moderate quality evidence; Agree: 73%, Mostly agree: 18%, Partially agree 9%, Disagree: 0%)

3. Method to ensure the quality of EGD for detection of GC

Most guidelines recommended that the stomach should be systematically observed with sufficient time (e.g., 7 min). To improve endoscopic visibility, the use of mucolytic or defoaming agents was recommended in some countries (Japan,^18^ Chile,^9^ and Mexico^19^).

The integrated statement in this category was;

- **The stomach should be systematically observed with sufficient time to detect GC**.

(Moderate quality evidence; Agree: 59%, Mostly agree: 33%, Partially agree: 8%, Disagree: 0%)

4. An individual GC risk assessment by EGD

The use of image‐enhanced endoscopy (IEE) was recommended to accurately detect and risk‐stratify gastric atrophy and GIM (UK,^15^ Chile,^9^ Mexico,^19^ and Europe^16^). Systematic biopsies (ex. updated Sydney system protocol^20^) were recommended in most non‐Asian countries.

The integrated statement in this category was;

- **In combination with white light endoscopy, IEE and biopsies should be considered for the assessment of chronic atrophic gastritis and GIM**.

(Moderate quality evidence; Agree: 42%, Mostly agree: 25%, Partially agree: 33%, Disagree: 0%)

5. Extraction of high‐risk patients of GC after EGD

Patients with endoscopic or histological diagnosis of GIM, and patients with gastric atrophy and/or GIM affecting both antral and corpus mucosa should be identified as they are considered to be at higher risk for GC (Europe^16^). Endoscopic findings of *H. pylori*‐negative status (which means “never infected”) and gastric mucosal atrophy are proposed for risk stratification (Japan^18^).

The integrated statement in this category was:

- **Extensive atrophy or presence of GIM should be identified by endoscopic findings or histology**.

(Moderate quality evidence; Agree: 90%, Mostly agree: 0%, Partially agree: 10%, Disagree: 0%)

6. Qualitative or differential diagnosis of GC by EGD

The use of high‐definition endoscopy with chromoendoscopy or IEE was recommended in most countries with a strong (Japan,^18^ Europe^16^) to moderate (South Korea,^13^ Mexico^19^) level of evidence. Chromoendoscopy or IEE and, where available, magnification endoscopy are ideal methods to determine the extent of early neoplastic lesions.

The integrated statement in this category was:

- **High‐definition endoscopy with chromoendoscopy, IEE, and where available, magnification endoscopy is recommended for the diagnosis of neoplastic lesions**.

(High quality evidence; Agree: 83%, Mostly agree: 17%, Partially agree: 0%, Disagree: 0%)

7. Endoscopic assessment to choose the therapeutic strategy for GC

Patients with an endoscopically visible lesion should undergo staging by white light endoscopy and histopathological diagnosis using biopsy specimens to determine the therapeutic strategy. A comprehensive diagnosis of lesion extent and depth, presence of an ulcer, and histology should be made. Endoscopic ultrasonography may be helpful in determining the depth of invasion of GC (Japan^18^ and Korea^13^).

The integrated statement in this category was:

- **Patients with an endoscopically visible lesion harboring dysplasia or carcinoma should undergo endoscopic staging and treatment**.

(Moderate quality evidence; Agree: 84%, Mostly agree: 8%, Partially agree: 8%, Disagree: 0%)

## DISCUSSION

According to the data available at GLOBOCAN 2020, the age‐standardized ratio per 100,000 for GC incidence and mortality in the world is 11.1 and 7.7, respectively.^1^ Among 35 countries with a higher incidence, and in 41 countries with higher mortality,^1^ only a few countries have guidelines for the management of GC. Establishing guidelines for screening and surveillance of patients who are at high risk of developing GC has the potential to diagnose and treat GC at an earlier stage and improve mortality from GC. In this study, we collected guidelines from various areas around the world including the very high and low incidence of GC. Essential and minimum recommendations regarding the screening and diagnosis of GC were extracted through a consensus of an international expert panel.

An effective method to narrow down the individuals who harbor gastric atrophy and GIM is needed. Examination for *H. pylori* serology combined with serum PG testing is a non‐invasive, low‐cost modality. A combination of low PG I or PG I/II ratio with negative *H. pylori* serology antibodies suggests the highest risk for GC.^21^ The ABCD method for the detection of individuals with high risk has been extensively investigated in GC high‐incidence areas. This method categorizes patients tested for *H. pylori* serology (HP) and PG I/II ratio (sPG) into low‐risk (A: HP−, sPG−), moderate‐risk (B: HP+ and sPG−), high‐risk (C: HP+ and sPG+) and very high‐risk (D: HP−, sPG+).^22^ A limitation of this method is that PG testing cannot be applied to individuals who had undergone *H. pylori* eradication, or those with several other conditions such as those who take proton‐pump inhibitors. Nevertheless, considering the low cost and handiness, initial screening with the ABCD method in areas with high to moderate‐incidence of GC would be feasible to extract individuals who have gastric atrophy or GIM.

Case‐control studies in a very high incidence of GC areas have reported that the odds ratio (OR) of GC mortality by introducing endoscopy screening was 0.206 to 0.695.^3^, ^23^, ^24^ Data from a national screening program in South Korea revealed that the number of endoscopic screening was related to OR of GC mortality; once: OR 0.60, twice: OR 0.32, and three or more times: OR 0.19.^3^ Although the effectiveness varies among esophageal cancer, non‐cardia GC and cardia GC, it has been reported that one‐time endoscopic screening program was effective in the prevention of all types of upper gastrointestinal cancer in individuals aged 40–69 years in high‐risk areas in China.^25^

To improve the quality of screening endoscopy, a systematic screening protocol is important. In Japan, where the incidence of GC is very high, an average of 40 images are often taken for thorough observation of the stomach in specialized centers. However, the number of images is not standardized in community‐based screening EGD, thus a basic observation method that requires at least 22 pictures to cover all areas of the stomach has been proposed.^26^ The alphanumeric coded upper gastrointestinal screening method has been effectively used in high prevalence GC countries such as Peru,^27^ Mexico,^28^ and Colombia.^29^ Moreover, the detection rate of early GC during screening endoscopy using white‐light imaging has been reported as 0.06% when examination time was shorter than 3 min,^30^ 0.2% when shorter than 5 min,^31^ and 0.9% when 7 min or longer.^32^ Although there may be differences in detection ability and examination time among experts and non‐experts, complete photodocumentation is recommended as an important quality parameter during screening endoscopy.^33^

Recently, methods for screening endoscopy are gradually changing. The use of transnasal thin endoscopy has increased due to technological advances in brightness, improved definition, and its thin caliber which is more acceptable for non‐sedative procedures. A retrospective comparison between transnasal thin endoscopy and transoral endoscopy reported that there were no significant differences in GC detection and proportion of early GC among detected lesions.^34^ On the other hand, the use of IEE has also increased during screening EGD. A multicenter randomized controlled trial reported that screening endoscopy by second‐generation narrow‐band imaging (NBI) had a similar detection rate of early GC but higher positive predictive value compared to surveillance by white light imaging (WLI) in a high‐risk population.^35^ Screening endoscopy using blue‐laser imaging^36^ or linked‐color imaging^37^ were reported to be better than WLI in regard to higher detection rates of neoplasms and reduced rates of overlooked neoplasms.

Endoscopic findings such as atrophy and GIM, nodularity, enlarged folds, and gastric xanthoma are associated with the risk of GC.^18^ The Sydney system requires two biopsies each from the antrum and corpus, and one from the incisura angularis to assess the degree of atrophy and GIM.^20^ In Europe, the use of an operative link on gastritis assessment,^38^ which comprehensively assesses the risk of GC using a combination of severities of histological atrophy of biopsy specimens from fixed points of the gastric antrum and corpus, and operative link on GIM assessment,^39^ which assesses the risk of GC based on the degree of histological intestinal metaplasia, instead of atrophy, have been proposed. In Japan, the Kimura‐Takemoto classification,^40^ which classifies the spread of endoscopic gastric mucosal atrophy in six categories (Closed‐type I II III and Open‐type I II III), is widely used. For the diagnosis of *H. pylori*‐infected stomach, the Kyoto Classification of Gastritis has been reported as a reliable method.^41^, ^42^, ^43^ For the detection and diagnosis of GIM, the use of IEE has been reported to be better than WLI in prospective studies performed in Europe, the USA, and the Asia‐Pacific region.^44^, ^45^, ^46^ The routine use of IEE has spread globally, allowing targeted instead of random biopsy samples, and may also allow real‐time diagnosis of GIM without biopsies.

Several prospective studies have reported the efficacy of magnification endoscopy with NBI during screening or surveillance endoscopy in a real‐time setting.^47^, ^48^, ^49^, ^50^, ^51^, ^52^ For newly detected suspicious lesions, the accuracy of diagnosis for GC was 88%–99%, sensitivity was 60%–93%, and specificity was 93%–100%.^47^, ^48^, ^49^, ^50^, ^51^, ^52^ A previous report showed that the use of magnification endoscopy improved positive predictive value for biopsy in routine EGD, which included many patients with surveillance EGD following endoscopic resection for early GC.^53^ Diagnosis using magnification endoscopy with NBI has become an essential diagnostic modality in pre‐treatment assessment in regards of characterization and to determine tumor extent.^54^, ^55^, ^56^, ^57^

Simultaneous and metachronous development of GCs is often encountered in patients with GC. In patients who had undergone endoscopic treatment for GC, the residual stomach is at high risk for developing metachronous GC. A multicenter retrospective cohort study reported that scheduled endoscopic surveillance among patients after endoscopic resection for early GC was effective in detecting new lesions in a treatable stage by endoscopic resection.^58^

The limitation of this study is that the collection of guidelines was based on a hand search by each WEO committee member. In addition, the level of evidence of these minimum elements was defined by the consensus using the Delphi method. In countries with a very high incidence of GC, updated versions of guidelines are available, with many statements for various aspects in detail, for example, the Japanese guideline has 19 statements for the endoscopic diagnosis of early GC. In countries with low to the moderate incidence of GC, it may be difficult to adopt such detailed guidelines from the beginning. To establish an effective screening program specialized for one's country, we have to consider multiple factors such as age distribution of the population, the incidence, and mortality of GC, the prevalence of *H. pylori* infection, penetration of *H. pylori* eradication, medical cost, and accessibility to EGD. The first step is to accumulate information regarding these factors and create a guideline suitable for the current situation of each country.^59^

In conclusion, this study summarized currently available guidelines to extract minimum elements for screening and diagnosis of GC. We hope that these minimum elements will be a guide to promote the elimination of GC among countries with a high incidence of GC who lack national guidelines or screening programs.

## CONFLICT OF INTEREST

Kazuki Sumiyama is a Deputy Editor‐in‐Chief of DEN Open. The other authors have no conflict of interest for this article.

## FUNDING INFORMATION

None.

## ETHICS STATEMENT

This study complied with the Declaration of Helsink

## Supporting information




**Supplement Table 1**. Raw data of statements regarding screening and diagnosis of gastric cancer.
