# Supplementary material for: Proposal of minimum elements for screening and diagnosis of gastric cancer by an international Delphi consensus
Source: DEN Open. 2022 Feb 24;2(1):e97. doi: 10.1002/deo2.97 (PMC9302051; doi:10.1002/deo2.97)
Supplement: Supplementary file 1 — Supplement Table 1. Raw data of statements regarding screening and diagnosis of gastric cancer. [file DEO2-2-e97-s001.docx]

Supplement Table1. Raw data of statements regarding screening and diagnosis of gastric cancer.

| **Category** | **Statement from current guidelines** | **Country or society & evidence level (A: High, B: moderate, C: low, D: very low)** |
| --- | --- | --- |
|  |  |  |
| **Extraction of high-risk patients of GC before EGD** | Several factors such as *H. pylori*, atrophy, hereditary disease, smoking are risk factors for GC. Other possible factors include diet, lifestyle preferences, EBV infection. | Japan: C |
|  | Patients with chronic AG or IM are at risk for gastric adenocarcinoma. | ESGE: A,BSG: A |
|  | The risk of GC can be stratified before EGD. A beneficial economic effect can be expected from this risk stratification. However, issues about the optimal method remain. | Japan: C |
|  | The risk of GC development depends on the presence of *H. pylori* infection and the presence, severity and extension of AG, IM and dysplasia. Using this information, it is possible to estimate the GC incidence and need of endoscopic follow-up. | Chile: B |
|  | In intermediate to high-risk regions, identification and surveillance of patients with precancerous gastric conditions is cost-effective. | ESGE: B |
|  | A combination of serum *H. pylori* antibody and serum PG may be useful for risk stratification of GC. However, false negative results can occur in cases of severe atrophy and past infection in *H. pylori* antibody titer measurement and cut-off value, interpretation of PG levels, and PG I/PG II ratio cut-off value. | Japan: C |
|  | We suggest endoscopic screening should be considered in individuals aged >50 years with multiple risk factors for GC (male, smokers, pernicious anemia) in particular, in those with a 1st-degree relative with GC. | BSG:C |
|  | Low PG I serum levels or/and low PG I/II ratio identify patients with advanced stages of atrophic gastritis and endoscopy is recommended for these patients, particularly if *H. pylori* serology is negative. | ESGE:B |
|  | It is suggested to look for AG, IM or dysplasia in all symptomatic patients over 40 years old, or having first grade relatives with GC. | Chile: B |
|  | Atrophy, intestinal metaplasia, and low-grade dysplasia in the gastric mucosa are risk factors for early GC, whereas high-grade dysplasia is a precursor lesion for the disease. | Mexico: B |
| **Patients who need surveillance of GC** | A surveillance endoscopic examination is recommended for patients with risk factors for GC(clinical and endoscopic findings) | Japan: B |
|  | HGD and invasive carcinoma should be regarded as the outcomes to be prevented when patients with chronic AG or IM are managed. | ESGE: B |
|  | AG, IM, and dysplasia are conditions / lesions associated to a progressive GC risk. A scheduled endoscopic follow-up increases the probability of detect early GC (secondary prevention). | Chile: B |
|  | The follow-up interval should be adjusted to the estimated risk of GC | Chile: C |
|  | For patients with mild to moderate atrophy restricted to the antrum, there is no evidence to recommend surveillance. | ESGE: B, Mexico: D |
|  | Patients with IM at a single location have a higher risk of GC. However, this increased risk does not justify surveillance in most cases, particularly if a high-quality endoscopy with biopsies has excluded advanced stages of AG. | ESGE: B |
|  | Patients with IM at a single location but with a family history of GC, or with incomplete IM, or with persistent *H. pylori* gastritis, endoscopic surveillance with chromoendoscopy and guided biopsy in 3 years' time may be considered. | ESGE: C, BSG: C |
|  | Patients with advanced stages of AG, should be followed up with a high-quality endoscopy every 3 years. | ESGE: C, BSG: C |
|  | Patients with advanced stages of AG and with a family history of GC may benefit from a more intensive follow-up (ex: every 1-2 years) | ESGE: C |
|  | Patients with mild to moderate AG, IM (OLGA/OLGIM I/II), and those with persistent *H. pylori* infection may undergo follow-up EGD every 3 years | Chile: C |
|  | Patients with autoimmune gastritis may benefit from endoscopic follow-up every 3-5 years. | ESGE: C |
|  | We recommend that a follow-up gastroscopy should be performed at 12 months after complete endoscopic excision of adenomas, then ongoing surveillance gastroscopy annually thereafter, when appropriate. | BSG: C |
|  | We recommend regular surveillance endoscopy every 6–12 month for patients who have had curative endoscopic resection of early GC based on absolute or expanded criteria for early detection of metachronous gastric cancer | Korea: C |
|  | First-degree relatives of patients with GC, even without demonstration of AG, IM or *H. pylori* infection, might undergo follow-up EGD every 5 years | Chile: C |
|  | It is suggested to withdraw from the endoscopic follow-up program those patients whose life expectancy, by age or associated comorbidity, is estimated to be less than 10 years | Chile: B |
|  | Screening is suggested in subjects with a first-degree relative with a history of GC. | Mexico: D |
| **Method to ensure quality of EGD for detection of GC** | The use of gastric peristalsis-inhibiting drugs should be considered in cases in which observation is difficult because of intense peristalsis. | Japan: D |
|  | The use of mucolytic agents to dissolve and remove the gastric mucosa and defoaming agents is strongly recommended because improved visibility of the mucosa leads to the detection of early gastric cancer. | Japan: D, Chile: C,  Mexico: C |
|  | Sedatives and analgesics may be used with caution for possible adverse reactions in subjects who have strong anxiety or in whom observation is difficult because of reflex or body movements. | Japan: D |
|  | The observation duration of the stomach is associated with the detection of early GC. The should be observed taking sufficient time. | Japan: D |
|  | The stomach should be systematically observed to detect early GC. | Japan: D, BSG: B |
|  | Specific training improves the diagnosis of early GC. Key aspects include a complete and systematic gastric examination and a targeted search for flat lesions. | Chile: B |
|  | A minimum examination time of 7 minutes is suggested. | BSG:B |
|  | The best available endoscope should be used | Chile: C |
|  | The use of high-resolution endoscopic equipment, with or without magnification, is preferred for optimal detection of early-stage lesion. | Mexico: C |
|  | Systematic biopsies (Sydney) are recommended at each screening endoscopy, to update the future risk of GC. Biopsies must be submitted unequivocally identified | Chile: B |
|  | To adequately evaluate gastritis, atrophy, or intestinal metaplasia due to *H. pylori*, endoscopic biopsy samples should be obtained according to the updated Sydney protocol. | Mexico: A |
|  | Endoscopic report should include pictures of any focal lesions and a systematic photographic record that confirms the complete gastric exploration. Both the protocol suggested by Emura (Systematic Alphanumeric-Coded endoscopy) or by Yao (Systematic screening protocol for the stomach) are endorsed | Chile: C |
| **Individual GC risk assessment by EGD** | The usefulness of image-enhanced endoscopy for the detection of early GC is under discussion. | Japan: D |
|  | First-time diagnostic EGD should include gastric biopsies both for *H. pylori* infection diagnosis and for identification of advanced stages of atrophic gastritis. | ESGE: B |
|  | GA and IM may be detectable by WLI, however, the accuracy is poor. Therefore, we do not recommend establishing a diagnosis or risk stratification using WLI alone. | BSG: B |
|  | Biopsies of at least 2 topographic sites should be taken and clearly labelled in 2 separate vials. Additional biopsy of visible neoplastic suspicious lesions should be taken. | ESGE: B |
|  | Diagnosis of premalignant lesions requires histology. The Sydney protocol is recommended. Biopsies must be sent separately in two jars. | Chile: B |
|  | Whenever available and after proper training, virtual chromoendoscopy, with or without magnification, should be used for the diagnosis of gastric precancerous conditions, by guiding biopsy for staging atrophic and metaplastic changes and by helping to target neoplastic lesions. | ESGE: B |
|  | The OLGA and OLGIM systems for diagnosing gastritis, mainly at stages III and IV, can be useful for categorizing the risk for progression to gastric cancer. | Mexico: C |
|  | We recommend IEE as the best imaging modality to accurately detect and risk-stratify GA and IM. | BSG: B |
|  | Identification of gastric focal lesions is feasible with WLI. Its characterization improves with optical magnification and chromoendoscopy (with stains or digital) | Chile: B |
|  | The basic technique is white light high-resolution endoscopy and indigo carmine chromoendoscopy. When available, the use of optical magnification and digital chromoendoscopy is recommended | Chile: B |
|  | Conventional WLI cannot detect or accurately characterize early GC, making the use of chromoendoscopy (e.g., with indigo carmine) or IEE technologies necessary. | Mexico: B |
|  | In patients with dysplasia in the absence of an endoscopically defined lesion immediate high-quality endoscopic reassessment with chromoendoscopy (virtual or dye-based) is recommended. If no lesion is detected in this high-quality endoscopy, biopsy for staging of gastritis (if not previously done) and endoscopic surveillance within 6 months (if HGD) to 12 months (if LGD) are recommended. | ESGE: C |
|  | We recommend that patients with non-visible, LGD should undergo a 2nd endoscopy with enhanced imaging and extensive biopsy sampling, followed by a repeat endoscopy within 1 year if no visible neoplasia is detected. If there is persistent, non-visible LGD, endoscopy should be repeated annually thereafter. | BSG:C, Chile: C,  Mexico: C |
|  | We recommend that patients with non-visible, HGD should undergo an immediate 2nd endoscopy with enhanced imaging and extensive biopsy sampling. We recommend ongoing surveillance at 6 monthly intervals for persistent, non-visible HGD. HGD should be discussed at the regional upper gastrointestinal cancer multidisciplinary team and referred to a clinician with the appropriate expertise. | BSG: C, Chile: B |
|  | The diagnosis of HGD in the absence of endoscopic lesion should be corroborated by a second expert pathologist, and if there is agreement, the patient should be referred to a specialized center. | Mexico: C |
|  | We recommend that endoscopic appearances on WLI suggestive of GA or IM require escalation to high-resolution IEE and, where available, magnification endoscopy. | BSG: C |
|  | We recommend that patients with image-enhanced features of chronic AG should undergo biopsy for confirmation of endoscopic diagnosis; biopsy are directed at mucosal sites within Sydney protocol areas where enhanced imaging discloses GIM. Biopsy samples should be collected in separate containers and labelled as either 'directed' or 'random' to corroborate endoscopic staging assessment. | BSG: C |
|  | We suggest that a baseline EGD with biopsy should be considered in individuals aged >50 years, with laboratory evidence of pernicious anemia, defined by Vitamin B12 deficiency and either positive gastric parietal cell or intrinsic factor antibodies. As GA affects the corpus in pernicious anemia, biopsy should be taken from the greater and lesser curves. | BSG: C |
|  | We recommend that the location and extent of GA and IM should be clearly documented with photographic evidence. Endoscopic grading should be documented as distal gastric or proximal gastric. | BSG: C |
|  | We recommend that the number of gastric polyps, location of polyps and size of the largest polyp should be clearly documented. | BSG: C |
|  | We recommend that gastric polyps other than fundic gland polyps should be biopsied for histopathological assessment. | BSG: C |
|  | We recommend that if adenomas or hyperplastic polyps are present, the background mucosa should be endoscopically assessed for GA, IM, *H. pylori* and synchronous neoplasia. | BSG: C |
|  | It is suggested to stage the GC risk according to the OLGA for AG and / or IM (OLGIM), which should be included in the histological report | Chile: B |
| **Extraction of high-risk patients of GC after EGD** | GA, IM, goose bumps, swelling of the plica, and gastric xanthoma are endoscopic findings related to the risk of GC. | Japan: B |
|  | Risk stratification of GC may be implemented based on endoscopic findings of *H. pylori* -negative status and gastric mucosal atrophy. Thus, risk stratification using these two items is proposed. | Japan: C |
|  | Histologically confirmed IM is the most reliable marker of atrophy in gastric mucosa | ESGE: A |
|  | Patients with advanced stages of gastritis, that is GA and/or IM affecting both antral and corpus mucosa, should be identified as they are considered to be at higher risk for gastric adenocarcinoma. | ESGE: B |
| **Qualitative or differential diagnosis of GC by EGD** | IEE is useful for the qualitative diagnosis of early GC; thus, its use is recommended. | Japan: A |
|  | High definition endoscopy with chromoendoscopy is better than high definition WLI alone for the diagnosis of gastric precancerous conditions and early neoplastic lesions. | ESGE: A |
|  | We recommend that endoscopic appearance on WLI of gastric dysplasia and early GC require escalation to IEE and, where available, magnification endoscopy. | BSG: C |
|  | We recommend chromoendoscopy/IEE to determine the extent of lesion before endoscopic treatment of early GC | Korea: B |
|  | Ideally, early GC margin determination is achieved through the use of image-enhanced endoscopes (e.g., blue-laser imaging or narrow band imaging digital chromoendoscopy, followed by chromoendoscopy (e.g., with 0.2%indigo carmine with/without 1.5% acetic acid). | Mexico: B |
| **Endoscopic assessment to choose the therapeutic strategy for GC** | A close pretreatment endoscopic examination is necessary for determining the therapeutic strategy in cases of early GC. | Japan: D |
|  | Diagnosis of the histologic type of GC should be performed comprehensively by endoscopic diagnosis and histopathological diagnosis using biopsy specimens. | Japan: D |
|  | Although a rough estimation of lesion size can be obtained by endoscopy, an endoscopic diagnosis should be made on the premise that the lesion size should finally by judged after obtaining histopathological findings of the resected specimen. | Japan: D |
|  | In principle, conventional WLI should be used for determining the depth of invasion of early GC. If this is difficult, EUS may be a useful adjunctive diagnostic tool. | Japan: C |
|  | EUS before endoscopic resection of early GC may be helpful in determining the depth of invasion in some patients with early GC. | Korea: B |
|  | In principle, conventional WLI should be used for determining the presence/absence of active ulcers and ulcer scars associated with early GC. | Japan: D |
|  | IEE is useful for diagnosing the extent of invasion. | Japan: B |
|  | Patients with an endoscopically visible lesion harboring LGD or HGD or carcinoma should undergo staging and treatment | ESGE: A |
|  | We recommend IEE as the best imaging modality to accurately diagnose and stage gastric dysplasia and early GC. | BSG:B |

GC: gastric cancer, EGD: esophagogastroduodenoscopy, EBV: Epstein-Barr Virus, AG: atrophic gastritis, IM: intestinal metaplasia, ESGE: European Society of Gastrointestinal Endoscopy, BSG: British society of gastroenterology, PG: pepsinogen, WLI: white-light imaging, IEE: image enhanced endoscopy, OLGA: Operative Link of Gastritis Assessment, OLGIM: Operative Link on Intestinal-Metaplasia Assessment, HGD: high-grade dysplasia, LGD: low-grade dysplasia, EUS: endoscopic ultrasonography
